# Natural Habitat and Wild Behaviors of the Dwarf Cuttlefish, *Ascarosepion bandense*

**DOI:** 10.1002/ece3.72001

**Published:** 2025-09-02

**Authors:** Connor J. Gibbons, Frederick A. Rubino, G. Thomas Barlow, Daniella Garcia‐Rosales, Noel Guevara, Boogs Rosales, Sukanya Aneja, Dana Elkis, Glenn Dalisay Mendoza, Jhomer Ilagan Demayo, Edgar Oliverio Atienza, Tessa G. Montague

**Affiliations:** ^1^ Department of Neuroscience, The Mortimer B. Zuckerman Mind Brain Behavior Institute Columbia University New York City New York USA; ^2^ Department of Biology, Whitehead Institute Massachusetts Institute of Technology Cambridge Massachusetts USA; ^3^ National Geographic Society Manila Philippines; ^4^ International League of Conservation Photographers Manila Philippines; ^5^ Interactive Telecommunications Program New York University New York City New York USA; ^6^ Crystal Blue Resort Mabini Batangas Philippines; ^7^ Howard Hughes Medical Institute, Columbia University New York City New York USA

**Keywords:** camouflage, cephalopod, color, coral reef, cuttlefish, social behavior, visual texture, wild behavior

## Abstract

The dwarf cuttlefish, *Ascarosepion bandense* (formerly *Sepia bandensis* ), is a coleoid cephalopod like octopus and squid, and an emerging model organism for scientific research. Dwarf cuttlefish can change the color, pattern, and texture of their skin in milliseconds to camouflage with their surroundings and communicate with conspecifics. Their skin displays are directly controlled by the brain. Thus, observing the skin provides a window into neural processes in the brain. Despite the popularity of dwarf cuttlefish in public aquariums and laboratory research, little is known about their natural habitat and behaviors in the wild. We conducted a field study in the Batangas region of the Philippines using underwater photography, videography, and environmental measurements. We generated an image bank of the natural features in the environment, characterized the change in color profile at different depths, and surveyed the population of dwarf cuttlefish in coral reefs and silty barren environments (muck), at a range of depths, during both the day and night. All dwarf cuttlefish sightings occurred after sunset, at depths of 6–12 m, and on coral reefs. The animals exhibited multiple camouflage strategies, including complex skin patterning and adhesion of sand to their skin, as well as social skin displays in the presence of fish. Notably, despite apparent colorblindness, dwarf cuttlefish produced skin patterns with vibrant colors not recorded in laboratory settings, with some instances of apparent color matching to their surroundings. These findings challenge our understanding of cephalopod visual perception and camouflage and highlight the importance of studying animal behavior in its natural context. Our image bank and behavioral data are freely available on the interactive web tool, Cuttlebase (www.cuttlebase.org).

## Graphical abstract

Gibbons et al. performed a field study to characterize the dwarf cuttlefish—a popular aquarium and research cephalopod—in its natural habitat. They observed dynamic camouflage, social behaviors, and use of an expanded color palette in wild animals compared to laboratory animals. All of the wild data is hosted on a web tool, cuttlebase.org

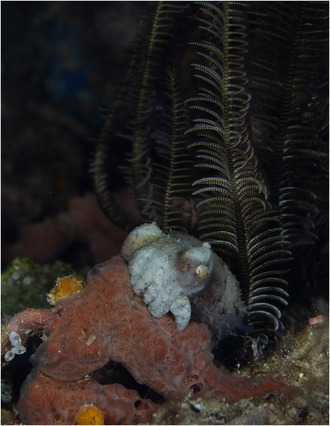

## 1 Introduction

The coleoid cephalopods—octopus, cuttlefish, and squid—are a class of marine mollusk with striking biological adaptations. They possess the largest brains of the invertebrates, three hearts, blue blood, ink sacs, regenerating arms, chemosensory suckers, jet propulsion, problem‐solving abilities, learning and memory, and neurally‐controlled skin capable of changing color, pattern, and texture to camouflage with the surroundings and communicate with other animals (Turchetti‐Maia et al. 2019; Fiorito et al. 1990; Richter et al. 2016; Reiter et al. 2018; Imperadore and Fiorito 2018; Kang et al. 2023; Montague 2023; Hanlon and Messenger 2018; Osorio et al. 2022; Shook et al. 2024). Cephalopods last shared a common ancestor with vertebrates over 600 million years ago (dos Reis et al. 2015) and Octopodiformes (octopuses) and Decapodiformes (squid and cuttlefish) diverged within Cephalopoda around 300 million years ago (Tanner et al. 2017). This early split from vertebrates, combined with their high biodiversity, makes cephalopods a compelling group for exploring questions bridging neuroscience, behavior, development, and evolutionary biology.

The dwarf cuttlefish (*Ascarosepion bandense*) is a cephalopod species that is well suited to laboratory research due to its small size (mantle length of < 90 mm), ability to be cultured for multiple generations in captivity, and short generation time (~4 months) (Montague et al. 2021). As a consequence, a growing collection of scientific tools for their study has been developed and are freely accessible via the web tool Cuttlebase (Montague et al. 2021, 2023; Lorig‐Roach et al. 2024). Dwarf cuttlefish exhibit rich behaviors, including dynamic camouflage, social skin patterns, and dynamic skin waves (Shook et al. 2024; Montague 2023), which are mediated by chromatophores—flexible sacs of pigment that are directly controlled by motor neurons projecting from the brain (Messenger 2001). Each skin pattern, therefore, is a visible representation of neural activity in the brain. During camouflage, cuttlefish recreate an approximation of what they see on their skin. Thus, camouflage patterns reflect the cuttlefish's perception of the visual world (Reiter and Laurent 2020; Montague 2023). Cuttlefish also engage their dynamic skin when interacting with potential mates, predators, and prey, using a series of innate and stereotyped skin patterns. These social skin patterns may therefore reflect a physical manifestation of internal state, such as aggression and fear (Shook et al. 2024). Leveraging the skin behaviors of cephalopods affords an opportunity to investigate how visual information or internal states are encoded in patterns of neural activity in the brain and transformed into a representation on the skin.

Dwarf cuttlefish are popular display animals in public aquariums, and there is increasing scientific interest in their biology. However, their ecology and wild behavior remain largely unexplored. Dwarf cuttlefish have been reported across the Indian Ocean, from Indonesia to the Marshall Islands (Jereb and Roper 2005; Norman and Reid 2000), but there are limited records on their depth, activity patterns, local habitats (e.g., reef or muck), and environmental conditions. Understanding their natural habitat is crucial both for improving husbandry practices and understanding the biological basis of camouflage and visual perception in this species. Coleoid cephalopods appear to have exceptionally high visual acuity (Nilsson et al. 2023; Temple et al. 2012; Sweeney et al. 2007). They independently evolved camera‐type eyes (Nilsson et al. 2023), and a significant portion of their brain appears to be dedicated to processing visual information: for instance, 75% of the dwarf cuttlefish brain volume is optic lobe (Montague et al. 2023). Despite these advanced visual capabilities and their elaborate camouflage, there is a conundrum: cuttlefish appear to be colorblind, possessing only a single opsin gene (Brown and Brown 1958; Chung and Marshall 2016), and failing color discrimination tasks (Brown and Brown 1958; Messenger 1977; Marshall and Messenger 1996; Mäthger et al. 2006). It is possible that color detection is unnecessary for cephalopods, as water absorbs longer wavelengths like red and orange, which limits the availability of color information underwater, and cephalopods possess polarization vision, which could offer additional visual information (Pungor and Niell 2023). Alternatively, color discrimination might be achieved using chromatic aberration (Stubbs and Stubbs 2016) or innate color pattern associations. However, the use of color has not been studied in wild dwarf cuttlefish.

Underwater scenes are rich in visual textures—defined as spatially homogeneous surfaces with repeating elements and stochastic variation—such as sand and seaweed (Portilla and Simoncelli 2000). During camouflage, cuttlefish generate high‐dimensional skin patterns to blend in with their surroundings (Woo et al. 2023). Rather than controlling each of their thousands to millions of chromatophores (Hanlon and Messenger 1988) independently, cuttlefish control groups of chromatophores to generate distinct pattern components, such as spots and stripes. These, in turn, are assembled into global skin patterns that approximate the surroundings (Hanlon and Messenger 1988; Reiter et al. 2018; Osorio et al. 2022). Each cuttlefish species has a unique repertoire of pattern components and skin patterns, which may have evolved to reflect the visual features of the local environment. Characterizing the visual statistics of the dwarf cuttlefish's natural habitat is therefore essential for understanding both the visual processing (input) and pattern generation (output) of the camouflage sensorimotor transformation.

**FIGURE 1:**
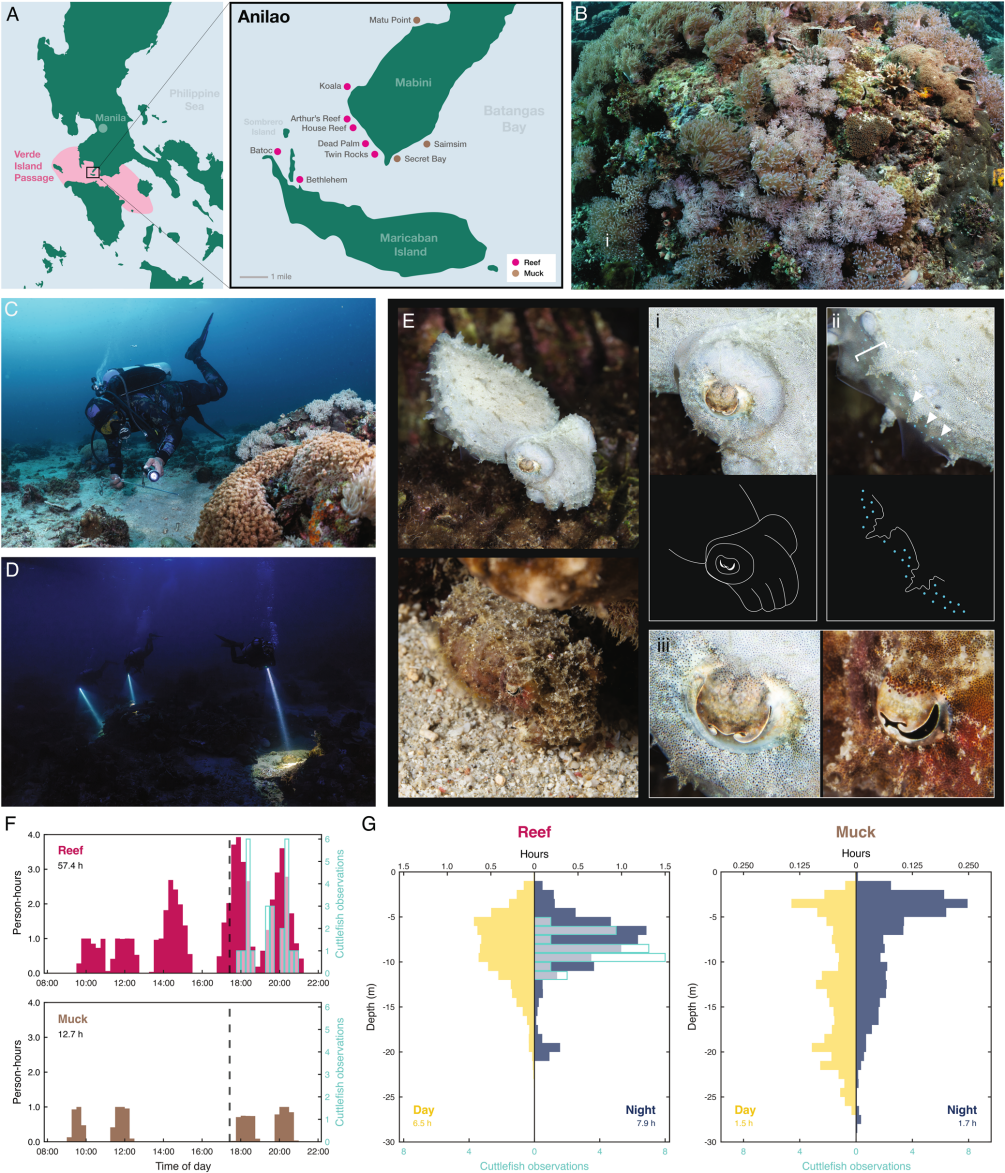
Dwarf cuttlefish are found in shallow coral reefs after dark. (A) Diving was conducted in multiple coral reef sites (pink) and muck sites (brown) in Anilao, a region within Batangas, Philippines that is positioned within the Verde Island Passage, one of the most biodiverse areas on Earth. (B) The coral reefs in Anilao are rich in biodiversity and include many soft and hard corals. (C) The search for dwarf cuttlefish was conducted by a team that included three professional spotters. (D) The search for dwarf cuttlefish was also performed after dark using flashlights. (E) Dwarf cuttlefish are identifiable by four distinctive features: (1) a bulbous ocular region (i); (2) three‐pronged papillae (ii); (3) blue iridescent spots on the fin (ii); and (4) a unique w‐shaped pupil that features a “serif” on the w (iii). (F) Total time spent by four spotters searching reef sites (pink, 57.4 h) and muck sites (brown, 12.7 h). Cuttlefish observations shown in aqua blue. Dotted line, sunset. (G) Average time spent by each spotter searching the indicated depths during the day (yellow) and night (dark blue) in reef sites (left) and muck sites (right).

To understand the wild habitat and behaviors of the dwarf cuttlefish, we conducted a field study in the Anilao region of Batangas, Philippines (Figure 1A). Anilao is positioned within the Verde Island Passage—one of the most biodiverse marine areas on Earth—and is home to a large array of cryptic and colorful marine organisms, including nudibranchs, frogfish, octopus, and cuttlefish (Carpenter and Springer 2005). We observed 25 dwarf cuttlefish, all of which were found on the coral reef after dark. The animals utilized complex skin patterns, three‐dimensional texture, and a vivid color palette for camouflage and social skin displays.

## 2 Results and Discussion

We conducted 8 days of scuba diving (16 dives; 70.1 dedicated spotter search hours) in Anilao, Philippines (Figure 1A). The coral reefs in Anilao are rich in biodiversity, with both soft (Figure 1B) and stony corals. There are also “muck” sites—barren areas with mud‐like substrate and some reef rubble or anthropogenic litter. Before initiating each dive, we performed a series of measurements to characterize the local environment and conditions at the dive site, including GPS coordinates of entry, visibility, surface temperature, wind speed, salinity, and water temperature (Table A1). In addition, we synchronized our dive computers with our photo and video equipment to log the time and depth of each piece of data (see Methods). The average ocean temperature and salinity were 29.6°C and 32.6 PSU, respectively, with little variation at the depths sampled (Table A1).

### 2.1 Habitat and Activity Pattern

**FIGURE 2:**
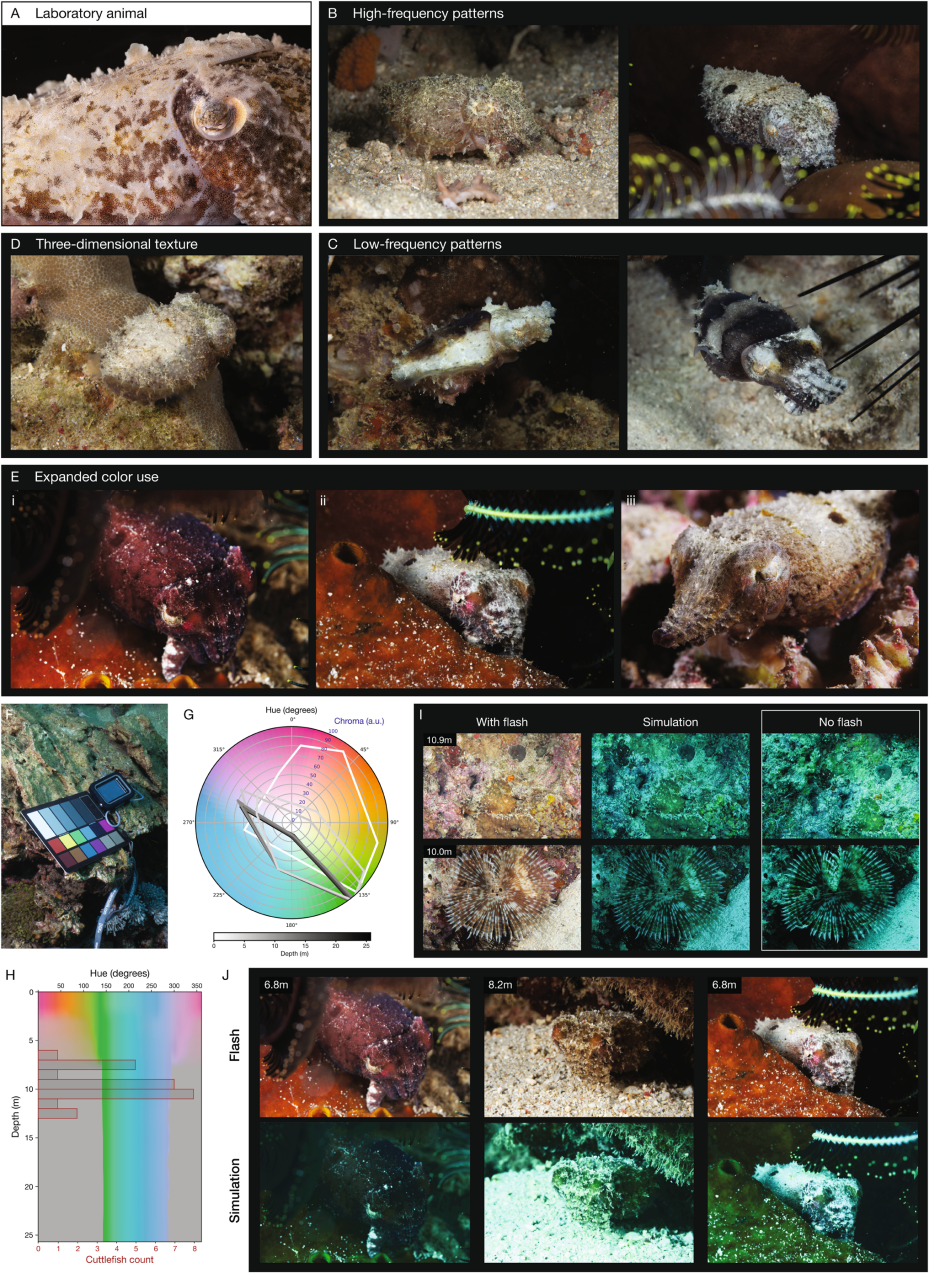
Dwarf cuttlefish produce vivid color displays at night. (A) Laboratory animals create skin patterns using primarily brown and yellow chromatophores, which lie above a white leucophore skin layer. (B–D) Wild cuttlefish adopted skin displays with high‐frequency patterns (B), low‐frequency patterns (C), and three‐dimensional texture (D). (E) Wild cuttlefish created vibrant skin patterns using chromatophores with a pink appearance (i,ii, Figure A1v,vi), and the appearance of rainbow colors around the eye (iii, Figure A1iv). (iii) is a frame from Video 2. (F) A color card was photographed at the water surface and at 10 depths to measure the change in color spectrum underwater. (G) A hue‐chroma color spectrum was calculated using the color cards photographed at different depths (see Methods). Each polygon contains the hue (color) and chroma (intensity) spectrum at a corresponding depth (white = surface to black = 25.7 m). Hue, degrees; chroma, arbitrary units (a.u.). (H) Plot of the color spectrum at different depths overlaid with cuttlefish observations (red). (I) Underwater images taken with flash (left column); color simulation to approximate the light profile at the specific depth of the photograph (middle column); no‐flash images in the same sequence (right column). (J) Cuttlefish images taken with flash (top row) and simulated to approximate the color profile at the specific depth of the photograph (bottom row).

Dwarf cuttlefish are active during both the day and night in the laboratory, but there are few reports of their activity patterns in the wild (Adam 1939; Norman and Reid 2000; Jereb and Roper 2005), which has important implications for designing appropriate stimuli and analyzing behavior. To locate dwarf cuttlefish, our team—which included three professional animal spotters—searched the reefs and muck using flashlights during daylight (Figure 1C) and after sunset (Figure 1D). Juvenile broadclub cuttlefish are commonly misidentified as dwarf cuttlefish. Therefore, we used four criteria to identify the species: (1) the shape of the ocular region, which is more bulbous in dwarf cuttlefish than in other local species (Figure 1Ei); (2) the longitudinal skin papillae, adjacent to the fin, which have a three‐pronged structure (Figure 1Eii); (3) the presence of blue iridescent spots scattered throughout the fin (Figure 1Eii); and (4) the w‐shaped pupil, which is unique among cuttlefish, featuring a serif on the “w” that is not found in other local species (Figure 1Eiii). During 70.1 h of searching conducted by spotters on reef (57.4 h) and muck (12.7 h) sites, both during the day (32.0 h) and night (38.2 h), from 0 to 28 m, we identified 25 dwarf cuttlefish. All 25 individuals were found in reef sites (Figure 1F), typically in crevices and caves, at 6–12 m depths (Figure 1G), and exclusively after sunset (Figure 1G). No animals were observed during daylight dives (20.9 animals expected from the nighttime reef discovery rate, *p* = 2.47 × 10^−7^, exact Poisson rate test) or on muck sites (expected value of 5.5 animals, *p* = 0.00681). Cuttlefish were observed in close proximity to a diverse array of marine organisms, including soft and stony corals, sponges, tunicates, annelid and polychaete worms, nudibranchs, urchins, sea cucumbers, crinoids, small shrimp, and fish. Notably, some individuals navigated effortlessly through the venomous spines of *Diadema* urchins (Figure 2C) and the adhesive pinnules of crinoids (Video 1). This suggests that they can avoid envenomation and entanglement, respectively, which could provide a defensive advantage against predation. Additionally, many dwarf cuttlefish were observed sharing caves or crevices with triggerfish, highlighting the ecological complexity of their natural environment. Together, this suggests that dwarf cuttlefish in this region live in complex reef environments at shallow depths and are active in the dark.

**VIDEO 1:** A dwarf cuttlefish perches, “walks” using its ventral arms, and passes through crinoids. Video content can be viewed at https://onlinelibrary.wiley.com/doi/10.1002/ece3.72001.

**VIDEO 2:** A dwarf cuttlefish displays vibrant colors around its eyes. Video content can be viewed at https://onlinelibrary.wiley.com/doi/10.1002/ece3.72001.

### 2.2 Skin Pigmentation

**FIGURE A1:**
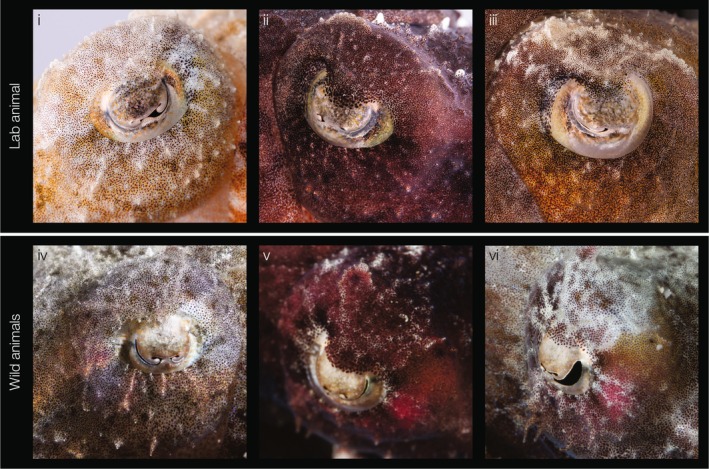
(i–iii) Enlarged view of the eye of a laboratory animal. (iv) Enlarged view of the eye of a wild individual (from Video 2); (v, vi) Eye of a separate wild individual.

Dwarf cuttlefish dynamically change the color and pattern of their skin by expanding and contracting hundreds of thousands of pigmented chromatophores that lie above a white leucophore skin layer. In the lab, dwarf cuttlefish predominantly utilize two chromatophore colors—yellow and brown—to create a range of hues (Figure 2A). Because all of the wild cuttlefish were observed after sunset, we used either flash photography or videography with a constant light source to record animal behavior. We observed dwarf cuttlefish employing multiple skin patterns in the wild, including the use of high‐frequency (Figure 2B), low‐frequency (Figure 2C), and three‐dimensional textural components (Figure 2D). Unexpectedly, under flash photography, wild dwarf cuttlefish displayed vibrant, multicolor skin patterns—apparent oranges, reds, and pinks—primarily around the eyes and anterior mantle (Figure 2E and Figure A1). These colors superficially resembled some of the surrounding reef organisms, such as coralline algae, encrusting corals, and sponges, and are not typically seen in laboratory animals housed in less complex environments and under LED lights (Figure A1).

Given that color is lost with increasing water depth, we sought to characterize the color spectrum at the depths inhabited by dwarf cuttlefish. We photographed a color card at the water surface and at a range of depths (Figure 2F) and then plotted the change in color spectrum underwater (Figure 2G) and overlaid it with the depths of cuttlefish sightings (Figure 2H). As expected, red light was rapidly attenuated underwater between 0 and 7 m, and the color space was compressed and translated towards a blue/green spectrum. Given that dwarf cuttlefish were only found after dark, we used an artificial light source to visualize them—reintroducing the full color spectrum to the subjects. To simulate the cuttlefish's appearance without artificial illumination, we used our color card data to create a model of color attenuation at depth (see Methods), which we tested on daylight underwater images taken with flash and compared to ground‐truth images taken without flash (Figure 2I). We then applied this model to our images of cuttlefish to simulate the appearance of the animals in ambient light at depth (Figure 2J). These data show that despite their relatively shallow dwellings and use of vibrant skin colors, dwarf cuttlefish were found at depths in which almost all wavelengths of light are filtered out except green and blue.

The observation that wild animals created skin displays using colors not seen in laboratory animals raises several questions, especially given that the colors were observed under conditions where much of the color spectrum is normally absent. It is possible that dietary differences between wild and captive populations influence pigment availability, but evidence to support this hypothesis is lacking. Alternatively, aspects of the complex natural environment—such as light availability, spectral composition, or behavioral context—could modulate chromatophore development and usage differently between wild and captive populations. Interestingly, juvenile dwarf cuttlefish in the lab briefly exhibit pink coloration around their eyes when they are 6–10 weeks old, but this pigmentation does not persist into adulthood. Long‐term chromatophore tracking in European cuttlefish ( *Sepia officinalis* ) has revealed that nascent chromatophores initially appear yellow, before progressing to orange, red, and eventually brown over the course of several days (Packard 1990; Reiter et al. 2018). It is possible that pink appears as an intermediate stage in dwarf cuttlefish chromatophore maturation.

Most research on cephalopod skin patterning over the last few decades has focused on a small number of species, yet even within this narrow scope, multiple variations in chromatophore organization have been observed. For instance, in *Octopus vulgaris* *sensu stricto*, the yellow chromatophores form the deepest chromatophore layer and the dark chromatophores are most superficial (Messenger 2001; Packard and Hochberg 1977), whereas in *Sepia officinalis* , this arrangement is reversed (Deravi et al. 2014). Notably, differences in chromatophore composition are also evident between more closely related taxa. Within the family Loliginidae, *Alloteuthis subulata* possesses only yellow and red chromatophores (Cornwell et al. 1997) while *Doryteuthis pealeii* has yellow, red, and brown chromatophores (Bower et al. 2024). It is plausible that *Sepia officinalis* and *Ascarosepion bandense*, which share a comparable taxonomic relationship, might also have evolved different color palettes. A recent reexamination of the Sepiidae lineages (Lupše et al. 2023) highlights the importance of expanding the exploration of cuttlefish species, especially those endemic to the Indo‐Pacific, where species diversity exceeds that of the Atlantic (Rosa et al. 2019).

### 2.3 Camouflage Behavior

**FIGURE 3:**
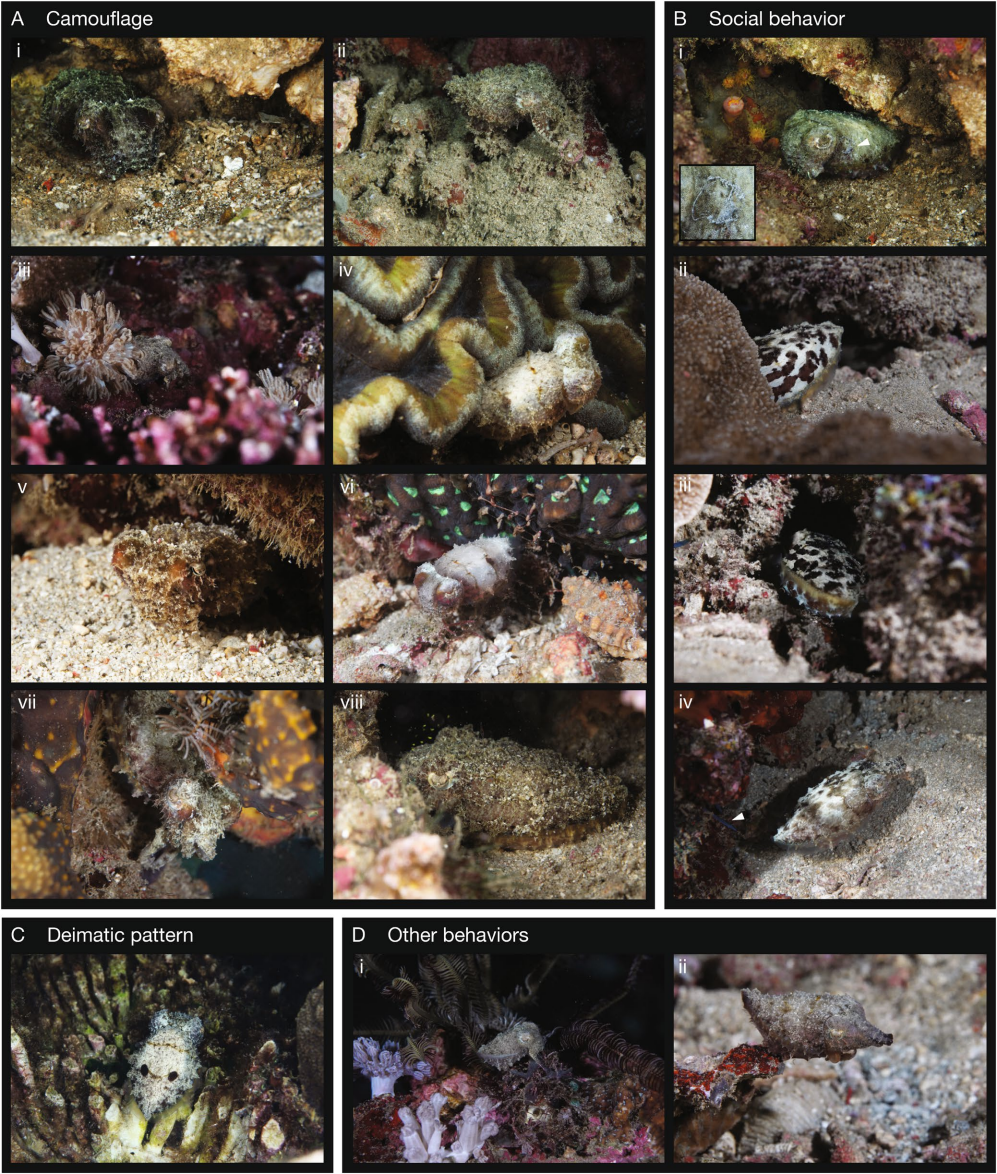
Dwarf cuttlefish use multiple camouflage strategies and adopt social skin patterns in the wild. (A) Dwarf cuttlefish utilize changes in color, pattern, three‐dimensional texture, and posture to camouflage. All images are photographs captured after dark using flash, except (vi) and (vii), which are frames from videos captured using a constant light source. (B) Evidence of social behavior in the wild. (i) A circular scar indicates a cuttlefish bite mark (white arrowhead). Inset: Enlarged view of the scar. See Figure A2 for a cuttlefish bite mark scar in the laboratory. (ii, iii) “Leopard” skin pattern examples (video frames). This pattern is normally observed in the laboratory in the presence of conspecifics (Figure A3). (iv) Half leopard pattern shown on the animal's left side when passing a trigger fish (blue tail, white arrowhead). Frame from Video 4. (C) A dwarf cuttlefish displaying a deimatic pattern—a pale skin pattern with two false eye spots on the mantle. (D) Additional dwarf cuttlefish behaviors observed. (i) Perching and “walking” using the ventral arms (Video 1); (ii) a possible hunting behavior, featuring a raised mantle and pointed arms with darkened tips (Video 5).

We observed dwarf cuttlefish adopting different camouflage strategies in the wild. Many animals utilized skin patterns incorporating visual textures, including instances where they appeared to mimic the visual texture of coral and reef surfaces (Figure 3Ai,ii,v,vii,viii). Most animals utilized their subcutaneous muscular papillae to create a three‐dimensional texture that effectively blended them with the spatial environment (Figure 3Ai,ii,v,vii), while some also adopted different body postures (Figure 3A) or adhered sand to their skin (Figure 3Aviii). Notably, we observed some cuttlefish using their expanded color palette during camouflage. One animal appeared to mimic the appearance of a yellow‐green coral on its eye (Figure 3Aiv); another adopted an orange‐red appearance near a red surface (Figure 3Av), and an additional animal created a pink skin pattern near pink coralline algae (Figure 3Avi). Although some color matching has been observed in both laboratory (Chiao et al. 2011) and wild (Akkaynak et al. 2013) *Sepia officinalis* , this is unexpected behavior for an animal that is thought to be colorblind. Is the color matching we observed coincidental, or do dwarf cuttlefish possess an unidentified mechanism to determine color? One theoretical model posits that cephalopods use their unusual pupil shape to discriminate color via chromatic aberration—a mechanism in which light of different wavelengths is focused at different distances from the lens (Stubbs and Stubbs 2016). Alternatively, some cephalopods may have evolved an innate associative camouflage strategy, in which they match certain chromatophore responses to specific reef organisms, even without direct color perception. However, these hypotheses remain speculative, and testing them currently presents experimental challenges.

Exposure to the high biodiversity and varied abiotic features of the coral reef appeared to induce more complex camouflage behaviors than we typically observe in laboratory animals. Interestingly, flamboyant cuttlefish (*Ascarosepion pfefferi*), which also inhabit this region, utilize a similar color palette in captivity and in the wild (Hanlon and McManus 2020), yet exhibit markedly different behaviors. Wild flamboyant cuttlefish are highly cryptic and spend their time on the seafloor; whereas in captivity, they more frequently display their vibrant colors and spend more time swimming through the water column (Hanlon and McManus 2020). Understanding the most ecologically relevant features of an animal's native environment will be essential for designing effective neuroethological studies and improving animal husbandry.

Our inability to find dwarf cuttlefish during the day suggests that this species is nocturnal or crepuscular—an activity pattern that is common in many other cephalopod species (Hanlon and Messenger 2018). But this observation raises the question of where they were located during the day. We observed one juvenile dwarf cuttlefish burying itself in sand (Video 3), suggesting this could be an effective strategy to avoid detection. Alternatively, these animals may employ rich camouflage behaviors in already obscured and inaccessible locations deep within a reef. Recent evidence indicates that cephalopod camouflage imposes a high metabolic cost (Sonner and Onthank 2024), which is not surprising given that each of the thousands to millions of chromatophores in the cuttlefish's skin is under continuous muscular control (Cloney and Florey 1968). It is likely that a nocturnal hunting strategy is adaptive for evading visual predators, and could allow cuttlefish to use less elaborate skin displays to still achieve effective camouflage.

**VIDEO 3:** A dwarf cuttlefish buries itself in sand. Video content can be viewed at https://onlinelibrary.wiley.com/doi/10.1002/ece3.72001.

### 2.4 Social Behavior

**FIGURE A2:**
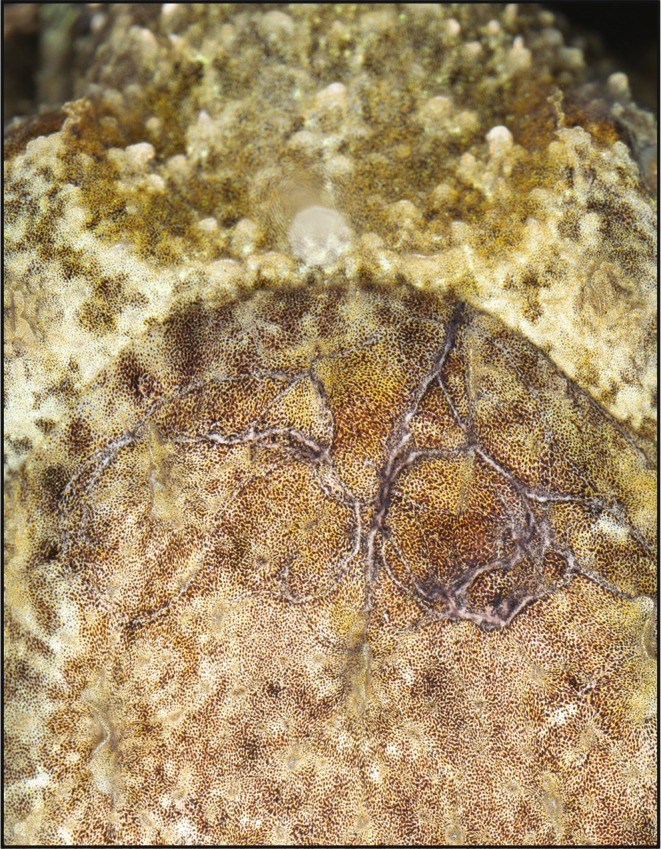
Circular scars on the skin of a laboratory dwarf cuttlefish who was bitten by another cuttlefish 5 days prior.

Some cephalopods exhibit rich and dynamic social skin behaviors (Hanlon and Messenger 2018; Shook et al. 2024). Dwarf cuttlefish use a series of discrete, stereotyped skin patterns during interactions with conspecifics in the lab, including during mating and male–male aggression (Montague et al. 2021; Shook et al. 2024). We did not observe any direct cuttlefish–cuttlefish interactions; however, some animals were found within 1 m of each other, suggesting they may regularly encounter conspecifics in the wild. Indeed, we identified two animals with potential cuttlefish bite mark scars—a common sign of intraspecific aggression (Figure 3Bi and Figure A2). In response to predators, many cephalopods adopt deimatic skin patterns, consisting of pale body coloration with two false eyespots on the mantle, which might serve as an alternative tactic to camouflage (Langridge et al. 2007). We observed multiple dwarf cuttlefish employing deimatic skin patterns, possibly in response to our human presence (Figure 3C).

**FIGURE A3:**
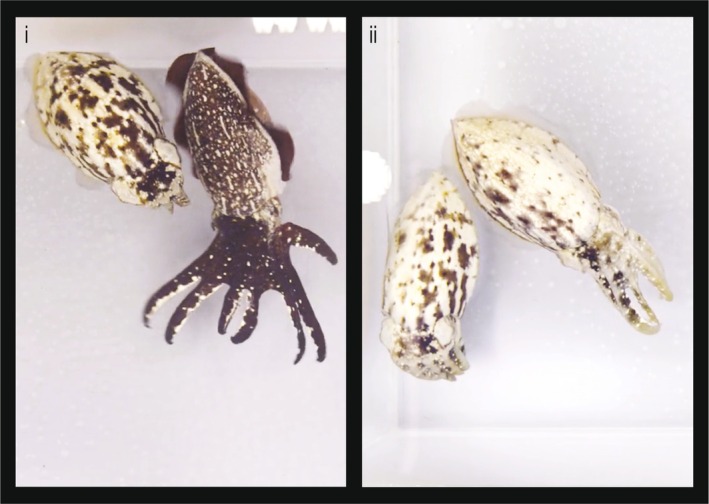
During laboratory interactions with conspecifics, dwarf cuttlefish may exhibit a stereotyped “leopard” skin pattern. (i) Leopard display (left animal); stereotypical “aggression” display (right animal). (ii) Both animals adopt a half leopard pattern on the side of the body that faces their opponent. All animals shown are male.

One of the common social skin patterns observed in the lab is a “leopard” pattern, consisting of large black and white patches, which we only see in the presence of other cuttlefish (Figure A3). It is sometimes used by females when being pursued by males, and by males during conflict, sometimes prior to an attack (Shook et al. 2024). Thus, we believe it may be used as a deterrent signal. We saw three instances of this pattern in the wild. On two occasions, the animal used the pattern when entering a cave (Figure 3Bii,iii). On the third instance, a cuttlefish carrying a crab claw within its arms flashed the leopard pattern on half of its body as it swam past a triggerfish in a cave (Figure 3Biv and Video 4). While anecdotal, this raises the possibility that cuttlefish use social skin patterns for both intra‐ and interspecific communication in the wild. At night, dwarf cuttlefish and triggerfish appeared to occupy the same areas within the reef. Although most triggerfish were observed sleeping, competition for food resources may still exist, potentially driving cuttlefish to preemptively engage in deterrent displays to avoid interference competition. While there is evidence of interspecific communication between octopuses and teleosts (Sampaio et al. 2024), further research is needed to determine whether predators, competitors, or prey respond to cuttlefish skin patterns, or if these displays evolved primarily for conspecific communication.

**VIDEO 4:** A dwarf cuttlefish, carrying a crab claw, produces a half leopard pattern on its left side when passing a trigger fish. Video content can be viewed at https://onlinelibrary.wiley.com/doi/10.1002/ece3.72001.

### 2.5 Other Behaviors

We observed a number of other interesting behaviors. A few dwarf cuttlefish used their arms to walk and perch on substrates (Figure 3Di and Videos 1 and 5). One cuttlefish traversed the reef with its arms extended, the tips of its arms darkened, and the posterior mantle raised, while it appeared to be hunting (Figure 3Dii and Video 5). This pattern could represent the anticipation of food or might be used to lure prey. Additionally, one animal created a strobing pattern on its skin as it was being followed by a diver (Video 6), suggesting this dynamic pattern may be a response to a perceived predator.

**VIDEO 5:** A possible hunting behavior, featuring a raised mantle and pointed arms with darkened tips. Video content can be viewed at https://onlinelibrary.wiley.com/doi/10.1002/ece3.72001.

**VIDEO 6:** A dynamic strobe skin pattern. Video content can be viewed at https://onlinelibrary.wiley.com/doi/10.1002/ece3.72001.

### 2.6 Visual Environment

**FIGURE 4:**
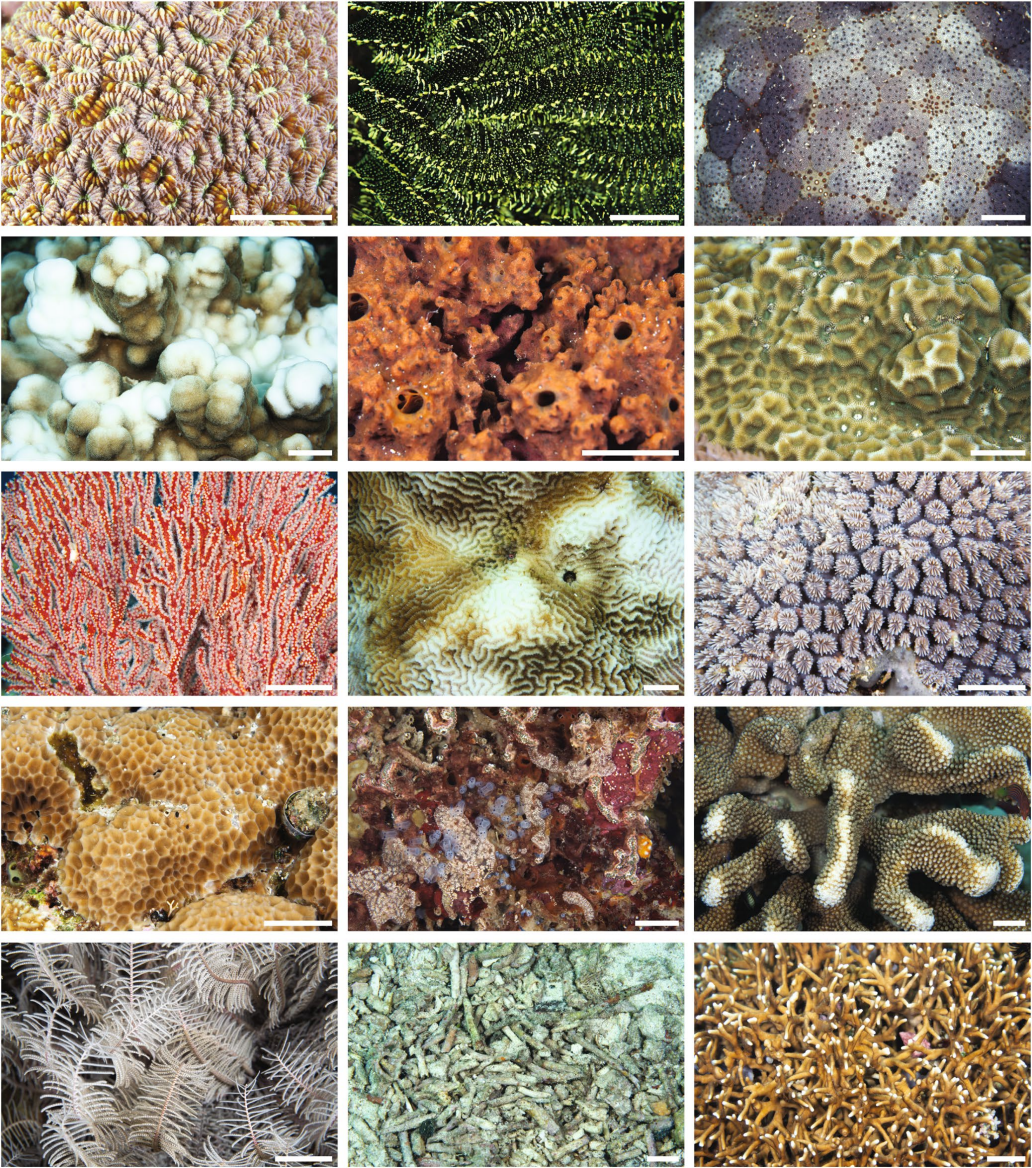
The natural habitat of the dwarf cuttlefish is rich in visual textures. Images captured using flash photography. For images captured without flash, see Figure A4. Scale bars, 20 mm.

**FIGURE A4:**
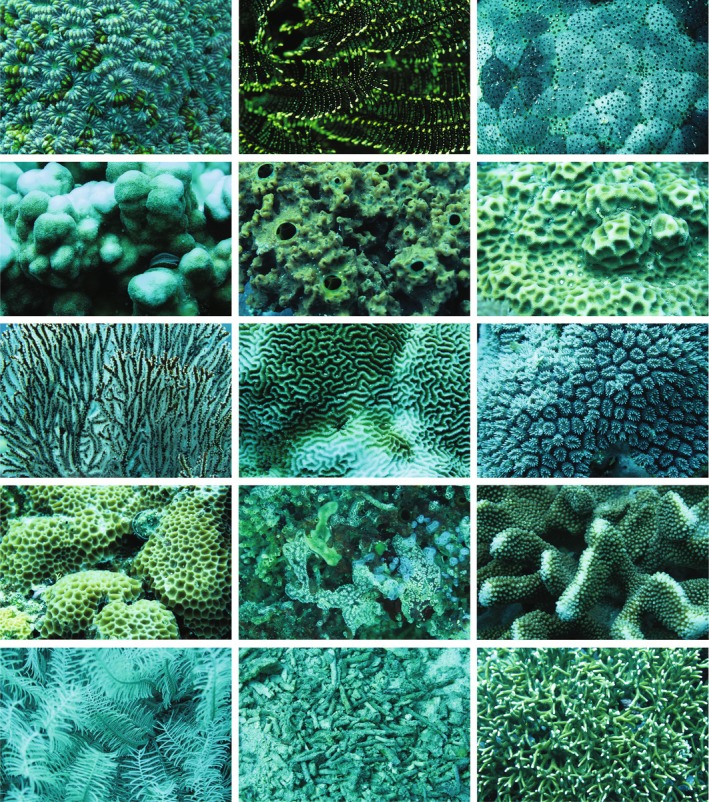
Visual textures captured without flash. For the same textures photographed with flash, see Figure 4.

All of the dwarf cuttlefish we observed in the wild were found in coral reef sites. We therefore photographed the sand, gravel, corals, crinoids, and sponges at each dive site with and without flash to create an image bank of 90 of the visual textures in this habitat (Figure 4 and Figure A4). To increase the utility of this dataset, we used a custom‐built laser system to measure 10 cm on each surface, allowing us to create a scale bar for every image (Figure 4). This entire image bank is freely available online (cuttlebase.org/downloads), and can be used for the analysis of aquatic scene statistics or the production of visual stimuli for laboratory behavioral studies.

Our survey of the underwater landscape reveals the high prevalence of visual textures—surfaces that are spatially homogenous with repeating elements and some random variation, including spots, stripes, speckles, and splotches (Portilla and Simoncelli 2000). Not surprisingly, the skin patterns of the dwarf cuttlefish also featured stripes, speckles, and splotches (Figures 2 and 3), which likely evolved to match the natural statistics of the animal's visual environment. This raises some interesting questions: Can we make predictions about what statistics might be represented in the cuttlefish brain to efficiently encode these visual textures? How do these visual textures compare to the sandy and muddy underwater environments in which *Sepia officinalis* , a well‐studied migratory cuttlefish species (Guerra 2006), evolved? Despite these textures existing in the dwarf cuttlefish habitat, the animal may not camouflage to all of them. Therefore, does the cuttlefish preferentially select substrates it can match? If so, how does it know what patterns it is capable of producing?

### 2.7 Limitations

There are several important limitations to acknowledge in this study. First, all observed dwarf cuttlefish were active exclusively at night, requiring the use of artificial light sources for documentation. The reintroduction of broad‐spectrum light revealed patterns and colors that may have been imperceptible under natural nocturnal conditions, and may be less visible under laboratory lighting conditions. Consequently, we cannot determine how these animals appeared in the absence of artificial illumination. Future studies could try to address this limitation by utilizing low‐light and infrared‐sensitive cameras to document cuttlefish without disrupting their natural visual environment. Additionally, deploying semi‐permanent underwater cameras for time‐lapse imaging could provide insights into cuttlefish behaviors in the absence of human influence. Second, these findings are based on a limited population, observed within a single season and restricted geographical range. Dwarf cuttlefish are distributed across the Indian Ocean, inhabiting some of the world's most biologically diverse marine ecosystems. Future research should investigate whether behavioral differences exist among populations due to environmental variation, ecological pressures, genetic divergence, or other factors.

### 2.8 Conclusion

This study provides new insights into the habitat and natural behaviors of the dwarf cuttlefish, raising broader questions about visual perception and camouflage in a supposedly colorblind animal. Future research should explore additional dive locations across the Indian Ocean to assess geographic, seasonal, and life‐stage variations. Additionally, incorporating biological sampling could improve our understanding of the genetic relationships among different dwarf cuttlefish populations, offering further context for their ecology, behavior, and camouflage strategies.

## 3 Methods

### 3.1 Locations and Diving

16 dives utilizing scuba were performed from November 11, 2024, to November 18, 2024. 18 days prior to the expedition, there was a typhoon, and on days 6 and 7 of the expedition, there was a second typhoon, which prevented the execution of some dives. The full moon was on November 15, 2024. Dives were conducted during the day and night, with 2–3 dives per day, each lasting for 50–80 min, and at depths ranging from 0 to 28 m. Diving was carried out using nitrox air with a 32% oxygen concentration to limit surface intervals between dives, and searching was conducted using flashlights. All 16 dives included the following dive team: three scientists, three professional spotters, and a photographer; furthermore, the first 13 dives included a videographer. A total of 138 h were spent underwater between all team members, with 70.1 h used by four spotters (CJG, EOA, GDM, JID) to actively search for cuttlefish.

Initial dive sites were chosen based on previous scouting efforts and verbal communication of dwarf cuttlefish sightings. Successful locations were revisited, and additional sites with different topographies, such as reef or muck, were added. Where possible, successful night diving sites were also selected during the day to establish if the cuttlefish had crepuscular or nocturnal behavioral adaptations.

### 3.2 Dive Log Analysis

All divers were equipped with Shearwater Peregrine (CJG, FAR, JID, EOA, TGM) or Perdix (BR, GDM, NG) dive computers. Each dive computer continuously measures the diver's depth to the EN13319‐specified accuracy of +1 m/−1.5 m, logged at 10‐s intervals, as stated by the manufacturer. Dive logs were synchronized to Anilao local time by comparison with https://time.is. Histograms for time of day (binned at 15‐min intervals) and depth (binned at 1 m intervals) used in Figure 1F,G were generated from spotter dive logs using MATLAB_R2024b software (The MathWorks Inc.). Only three (CJG, JID, GDM) of the four spotters' depth data could be made available due to a factory reset of one computer (EOA); therefore, Figure 1G was generated from the three available dive logs using the average time spent at each depth by these three spotters. The depth and time of cuttlefish sightings were documented manually by writing in underwater notebooks using measurements from the observer's dive computer and were later confirmed by cross‐referencing photograph and/or video evidence. Thus, each sighting was independently verified by at least two divers.

### 3.3 Environmental Measurements

The initial descent location at each dive site was recorded using a GPS (Garmin GPSMAP 79s). Visibility (m) was approximated using a Secchi disk (originally measured in feet). Conductivity (μS), temperature (°C), salinity (PSU), and depth (m) were measured using a SonTek CastAway‐CTD, and wind speed (kph) and air temperature (°C) were measured using a Kestrel 5000 Environmental Meter.

### 3.4 Cuttlefish Photography and Videography

Underwater photographs were captured using a Sony Alpha 1 camera with a Sony 90 mm macro lens in a Nauticam underwater housing, with SUPE D‐Pro strobes, and a SUPE RD95 Focus Light. Raw images were ingested using Photo Mechanic software, converted to JPEG in Adobe Lightroom, and brightness and contrast were adjusted in Adobe Photoshop. Underwater cuttlefish videos were captured using a Sony Alpha 1 camera with a 90 mm macro lens in Seacam housing with Keldan 4X 8000 lm 96 CRI video lights. Raw videos were downsampled, and brightness and contrast were adjusted in Adobe Premiere. Photographs and video files were synchronized to Anilao local time by comparison with https://time.is. Laboratory photographs were captured under an LED light panel (ikan Lyra LBX5 Bi‐Color LED Light Panel with DMX) using a Sigma fp L camera with a Sigma 105 mm f/2.8 DG DN macro lens. Images were acquired in RAW (DNG) mode and converted to JPEG using the Rawpy Python library. Raw and processed images are available to download from cuttlebase.org/downloads.

### 3.5 Visual Texture Photography

During daylight hours, at multiple different dive sites, photographs were captured of corals, sponges, sand, rubble, tunicates, and echinoderms using a Sony Alpha 1 camera with a Sony 90 mm macro lens in a Nauticam underwater housing, with SUPE D‐Pro strobes and a SUPE RD95 Focus Light. For each visual texture, one photograph was taken with flash and another without flash. A custom‐made laser system was built using two underwater lasers (OrcaTorch D570‐GL 2.01500 lm, green) mounted in parallel to maintain a fixed 10 cm separation between the beams, regardless of the distance from the photographer to the subject. For each visual texture, a photograph was taken with the lasers activated to facilitate scale bar calculation. On Day 4, one laser sustained saltwater damage. Thereafter, a reference object of known dimensions (a flashlight) was photographed with each visual texture to permit scale measurements. Raw images were converted into JPEG in Adobe Lightroom, and then brightness and contrast were adjusted in Adobe Photoshop to standardize the brightness for figures. To create a scale bar, each image was imported into Fiji (ImageJ) and the laser or object of fixed length was measured in pixels. The *set scale* function was used to convert pixels to millimeters, and a 20 mm scale bar was generated for each image. For the images with a visible laser beam, the laser light was removed in Photoshop by encircling the laser dot using the lasso tool and using the *generative fill* function to replace the patch. For the images taken with an object of fixed size, a corresponding image was taken without the object. All images used in Figure 4 were captured with an object of fixed size; therefore, the *generative fill* function was not used for these images. All 90 texture images (including those in Figure 4 and others), with and without laser removal, are available at cuttlebase.org/downloads.

### 3.6 Color Profile Measurements

Underwater photographs were taken with a Sony Alpha 1 camera in RAW (.ARW) format and synchronized with depth readings from the photographer's dive computer. On the first day of diving, a color card series (WDKK Waterproof Color Chart) was acquired at 11 depths from the surface to 25.7 m (0.0, 4.6, 6.0, 11.7, 11.8, 14.0, 17.2, 19.4, 21.1, 22.2, and 25.7 m). The raw images were read with the Rawpy Python library (camera white balance off, auto brightness off, gamma = 1), and the average color over each panel of the color card was extracted for each depth. To simulate ambient downwelling light for flash photographs taken at night, long wavelength information was removed in accordance with these measurements using a custom Python script (github.com/tbarlow99/Philippines‐expedition‐data‐analysis). For the hue and chroma plots, the raw color card photographs were read using the Rawpy library and directly converted from RGB to LAB space with the Skimage rgb2lab tool.

## Author Contributions

**Connor J. Gibbons:** conceptualization (equal), investigation (lead), writing – review and editing (lead). **Frederick A. Rubino:** formal analysis (equal), investigation (equal), methodology (equal), visualization (supporting), writing – review and editing (supporting). **G. Thomas Barlow:** formal analysis (equal), methodology (equal), visualization (supporting), writing – review and editing (equal). **Daniella Garcia‐Rosales:** conceptualization (equal), investigation (supporting), writing – review and editing (equal). **Noel Guevara:** investigation (equal), methodology (equal), project administration (equal). **Boogs Rosales:** investigation (equal). **Sukanya Aneja:** software (lead). **Dana Elkis:** software (equal). **Glenn Dalisay Mendoza:** investigation (equal), methodology (equal). **Jhomer Ilagan Demayo:** investigation (equal), methodology (equal). **Edgar Oliverio Atienza:** investigation (equal), methodology (equal). **Tessa G. Montague:** conceptualization (equal), investigation (equal), project administration (lead), supervision (lead), visualization (lead), writing – original draft (lead), writing – review and editing (equal).

## Conflicts of Interest

The authors declare no conflicts of interest.

## Data Availability

All of the data in this study is available for download through cuttlebase.org. The custom Python script for color profile measurements is available on GitHub (github.com/tbarlow99/Philippines‐expedition‐data‐analysis).

## Appendix A

**TABLE A1:** Measurements of environmental parameters at each dive site.

Date (2024)	Day	Dive	Dive site	GPS of entry	Time of entry	Visibility (m)	Min temp (°C)	Max temp (°C)	Average temp (°C)	Min cond (μS)	Max cond (μS)	Average cond (μS)	Min salinity (PSU)	Max salinity (PSU)	Average salinity (PSU)	Depth (m)	Windspeed (kph)	Surface temp (°C)
				Garmin GPS	Dive computer	Secchi disk	CastAway										Kestrel	
Nov 11	1	1	Saimsim	N 13 41.6419 E 120 54.5228	8:56 AM	6.1	28.95	28.97	28.96	53515	53890	53652	32.5	32.7	32.5	6.0	Error	29.6
		2	Secret Bay	N 13 41.1572 E 120 53.7044	11:18 AM	2.7	29.33	29.56	29.41	54146	54213	54181	32.5	32.6	32.6	2.5	Error	29.8
Nov 12	2	1	Koala	N 13 41.1573 E 120 53.7044	1:22 PM	7.9	29.36	30.14	29.46	54056	54927	54138	32.5	32.6	32.5	8.1	0	30.1
		2	Dead Palm	N 13 41.7198 E 120 53.0755	5:00 PM	9.1	29.54	29.6	29.56	53954	54062	54009	32.3	32.4	32.4	4.7	0	29.5
		3	Arthur's Reef	N 13 41.7143 E 120 53.0797	7:24 PM	Night	29.37	29.4	29.38	54099	54200	54145	32.6	32.6	32.6	7.3	0	Error
Nov 13	3	1	Twin Rocks	N 13 41.3870 E 120 53.4029	1:57 PM	4.9	30.03	30.41	30.18	54795	55166	54949	32.6	32.6	32.6	3.0	0	30.7
		2	Bethlehem	N 13 40.3737 E 120 50.4802	5:20 PM	9.1	29.68	30.01	29.82	54460	54817	54611	32.6	32.6	32.6	10.1	1.8	29.6
		3	Bethlehem	N 13 40.3681 E 120 50.4823	7:31 PM	Night	29.69	29.71	29.69	54477	54507	54489	32.6	32.6	32.6	10.9	1.3	28.0
Nov 14	4	1	Batoc	N 13 41.3870 E 120 53.4029	9:35 AM	8.5	29.61	29.63	29.62	54574	54599	54585	32.7	32.7	32.7	9.0	3.5	29.6
		2	Arthur's‐to‐House Reef	N 13 41.8379 E 120 49.5961	4:47 PM	6.4	29.48	29.67	29.62	54105	54258	54186	32.4	32.6	32.5	9.9	4.0	28.4
		3	Arthur's‐to‐House Reef	N 13 42.4612 E 120 52.4727	7:06 PM	Night	29.37	29.38	29.37	54245	54294	54275	32.7	32.7	32.7	9.4	1.3	28.9
Nov 15	5	1	Secret Bay	N 13 41.1608 E 120 53.7232	5:46 PM	Night	29.46	29.73	29.65	54499	54758	54670	32.7	32.8	32.8	4.4	4.2	28.7
		2	Arthur's‐to‐House Reef	N 13 42.4504 E 120 52.4907	8:04 PM	Night	29.4	29.6	29.54	54349	54417	54390	32.6	32.7	32.6	9.0	2.4	28.1
Nov 16	6		Typhoon															
Nov 17	7		Typhoon															
Nov 18	8	1	Bethlehem	N 13 42.4502 E 120 52.4900	11:26 AM	6.4	29.46	29.48	29.47	54329	54344	54337	32.7	32.7	32.7	8.5	1.9	31.3
		2	House Reef	N 13 42.4082 E 120 52.5235	5:36 PM	Night	Error	Error	Error	Error	Error	Error	Error	Error	Error	Error	9.2	29.5
		3	Matu Point	N 13 45.3172 E 120 54.3976	7:50 PM	Night	Error	Error	Error	Error	Error	Error	Error	Error	Error	Error	2.6	28.3
